# Novel Luteolin-Loaded Chitosan Decorated Nanoparticles for Brain-Targeting Delivery in a Sporadic Alzheimer’s Disease Mouse Model: Focus on Antioxidant, Anti-Inflammatory, and Amyloidogenic Pathways

**DOI:** 10.3390/pharmaceutics14051003

**Published:** 2022-05-06

**Authors:** Haidy Abbas, Nesrine S El Sayed, Nancy Abdel Hamid Abou Youssef, Passent M. E. Gaafar, Mohamed R. Mousa, Ahmed M. Fayez, Manal A Elsheikh

**Affiliations:** 1 Department of Pharmaceutics, Faculty of Pharmacy, Damanhour University, Damanhour 22511, Egypt; drmanalelsheikh87@gmail.com; 2 Department of Pharmacology and Toxicology, Faculty of Pharmacy, Cairo University, Giza11562, Egypt; 3 Department of Pharmaceutics, Faculty of Pharmacy, Pharos University in Alexandria (PUA), Alexandria 21500, Egypt; nancyabouyoussef@yahoo.com or; 4 Department of Pharmaceutics, Division of Pharmaceutical Sciences, College of Pharmacy, Arab Academy for Science, Technology and Maritime Transport, Alexandria 21500, Egypt; passent.ehab@aast.edu; 5 Department of Pathology, Faculty of Veterinary Medicine, Cairo University, Giza 12211, Egypt; mohamed.refat@cu.edu.eg; 6 Department of Pharmacology and Toxicology, School of Life and Medical Sciences, University of Hertfordshire Hosted by Global Academic Foundation, New Administrative Capital, Cairo 11835, Egypt; a.fayez@gaf.edu.eg

**Keywords:** Alzheimer’s disease, luteolin, cognitive dysfunction, B-amyloid, neuroinflammation, chitosomes

## Abstract

Preparation and evaluation of a non-invasive intranasal luteolin delivery for the management of cognitive dysfunction in Alzheimer’s disease (AD) using novel chitosan decorated nanoparticles. Development of luteolin-loaded chitosomes was followed by full in vitro characterization. In vivo efficacy was evaluated using a sporadic Alzheimer’s disease (SAD) animal model via intracerebroventricular injection of 3 mg/kg streptozotocin (ICV-STZ). Treatment groups of luteolin suspension and chitosomes (50 mg/kg) were then intranasally administered after 5 h of ICV-STZ followed by everyday administration for 21 consecutive days. Behavioral, histological, immunohistochemical, and biochemical studies were conducted. Chitosomes yielded promising quality attributes in terms of particle size (PS) (412.8 ± 3.28 nm), polydispersity index (PDI) (0.378 ± 0.07), Zeta potential (ZP) (37.4 ± 2.13 mv), and percentage entrapment efficiency (EE%) (86.6 ± 2.05%). Behavioral findings showed obvious improvement in the acquisition of short-term and long-term spatial memory. Furthermore, histological evaluation revealed an increased neuronal survival rate with a reduction in the number of amyloid plaques. Biochemical results showed improved antioxidant effects and reduced pro-inflammatory mediators’ levels. In addition, a suppression by half was observed in the levels of both Aβ aggregation and hyperphosphorylated-tau protein in comparison to the model control group which in turn confirmed the capability of luteolin-loaded chitosomes (LUT-CHS) in attenuating the pathological changes of AD. The prepared nanoparticles are considered a promising safe, effective, and non-invasive nanodelivery system that improves cognitive function in SAD albino mice as opposed to luteolin suspension.

## 1. Introduction

Alzheimer’s disease (AD) is often characterized by the advanced deterioration of cognition and memory, and it accounts more than 60% of most dementia cases. The most common form of AD among the elderly is the Sporadic Alzheimer’s disease (SAD) [1,2]. The presence of oxidative stress markers is one of the earliest changes that occurs in AD brains. Then, it proceeds to accumulate neurofibrillary tangles and consequently, the appearance of amyloid deposits. In AD brains, the tau proteins and solid amyloid-β (Aβ) assemble into amyloid-like filaments called tangles and plaques, respectively [3]. To date, the pharmacological management of AD has been limited to medications or supplements, which have not been proven to decrease AD risk [4]. Unfortunately, currently available treatments for AD that are approved by the Food and Drug Administration (FDA) only offer symptomatic relief and are not able to delay or cure the disease. This involves the use of cholinesterase inhibitors such as galantamine, donepezil, tacrine, and rivastigmine. Recently, it was hypothesized that antioxidants may be useful for the treatment of various memory-related diseases. Nevertheless, there is a need for advanced, highly tolerated, and more efficacious treatments [4].

Phytotherapeutics recently gained an enormous popularity because they often exhibit reduced toxicity and diverse therapeutic efficacies. Luteolin (LUT) (Supplementary Figure S1) is a naturally occurring, yellow crystalline flavonoid found in various types of vegetables and fruits. It is a microcrystalline powder that is sparingly soluble in water and in organic solvents. LUT is regarded as one of the most important bioactive flavonoids with potential pharmaceutical applications [5]. In traditional Chinese remedies, LUT-rich plants have been used for treating various diseases, such as inflammatory disorders, hypertension, and cancer. In the last two decades, studies on LUT have revealed its therapeutic potential in reducing AD symptoms in many in vitro and in vivo study models. The neuroprotective effect of luteolin has been attributed to its antioxidant and anti-inflammatory properties through modulation of transcription factors and inhibition of various protein kinases [6,7]. Nevertheless, LUT suffers from some drawbacks such as poor solubility in water, extensive first-pass metabolism, and a low oral bioavailability. In addition, it was also found to exhibit low permeability through the blood–brain barrier (BBB), which then inhibits an effective and sustained drug concentration at the target organ [8,9].

Over the past years, various tactics have been developed to improve LUT solubility and hence the dissolution rate [10]. These include salt formation, prodrug analogs, nano-solubilization through particle size reduction (e.g., nanosuspension, and nanoemulsion) [11] and complex solubilization (e.g., use of surfactants, phospholipids, and cyclodextrins) [5,12,13,14].

Many challenges exist that hamper drug targeting to the brain through either non-invasive enteral or invasive parenteral routes of administration [15]. Therefore, exploiting the intranasal route offers many advantages over both oral and parenteral routes including non-invasiveness, no need for sterile preparation, self-patient administration, shorter onset of action, and higher brain exposure by circumventing the intestinal/hepatic first-pass metabolism and bypassing the BBB [4,15,16,17]. Consequently, the intranasal route represents a promising route for direct non-invasive brain delivery, especially for diseases that require chronic treatment, such as AD. Nevertheless, some limitations may hamper efficiency in drug delivery, such as limited absorption capacity (maximum of 1% of the oral dose) [15]. Besides, the solubility of the drug at such a small dose volume is an important requirement. In addition, the rapid clearance rate inside the nasal cavity lessens the time needed for drug dissolution before being absorbed [4,16]. Therefore, there is a need to encapsulate the drug inside the nanocarrier which possesses a mucoadhesive property.

Liposomes have been widely used as a drug delivery system for improving drug efficacy and eliminating drug-related toxicity or undesirable effects. However, conventional liposomes encounter many challenges associated with poor physical and chemical stability, loss of encapsulated cargo, and rapid elimination from blood circulation [18].

Surface modification of liposomes using versatile functional biopolymers has emerged as an attractive strategy to overcome these drawbacks [19].

Chitosan is a linear natural cationic polysaccharide frequently used as a coating material for liposomes [20].

At acidic pH, chitosan possesses a high density of positive charge due to the presence of amino groups, thus, it can bind to the anionic liposomes forming chitosan-decorated liposomes (chitosomes). Chitosomes possess good mucoadhesive property, enabling a prolonged retention time for the encapsulated bioactive compounds in the nasal mucosa due to the electrostatic interactions with the negatively charged endothelial surface of mucous membranes. It absorbs water from the surrounding mucus and swells, leading to the formation of a gel-like structure that is expected to remain in the nasal cavity for prolonged periods of time surmounting the mucociliary clearance and leading to improved drug absorption from nasal mucosa [21].

In addition, it exhibits good biocompatibility and biodegradability which affects its bioactivity on the blood–brain barrier at the molecular level, which is advantageous in therapies for neurological disorders [19,20,21,22]. Its hydrophilic nature makes it useful for biomedical applications with enhanced permeation effects [21,22].

Accordingly, the poor water solubility, poor membrane permeability, and the first-pass effect of the polyphenolic luteolin may be circumvented through the use of chitosomes that serve as a promising approach for drug delivery. This present study is the first to evaluate brain targeting of luteolin via intranasal chitosomes through the olfactory mucosa. The ultimate goal is to enhance luteolin-brain uptake while attaining a rapid onset of activity and efficacy at lower doses.

## 2. Materials and Methods

Lipoid^®^ S100 (l-α-phosphatidylcholine) (MW = 786.1 g/mol) and phosphatidylserine (MW = 385.304 g/mol) were purchased from Lipoid AG (Ludwigshafen, Germany) (Cholesterol was a kind gift from The Nile for Pharmaceuticals and Chemical Industries (Cairo, Egypt) (MW = 386.654 g/mol). Oleic acid (MW = 282.47 g/mol), luteolin (≥98% HPLC) was purchased from Baoji Guokang Bio-Technology Co., Ltd. China, and chitosan (ChitoClear^TM^) was purchased from the Primex BioChemicals AS (Avaldsnes, Norway) (low MW, 50,000–190,000 Da). Streptozotocin (STZ) was purchased from Sigma–Aldrich (St. Louis, MO, USA) catalog number (S0130). Other chemicals and reagents that are not previously specified were obtained from Sigma–Aldrich Chemical Co (St. Louis, MO, USA).

### 2.1. Preparation of Chitosomes (Chitosan-Coated Anionic Liposomes) (CS-NPs)

Anionic liposomes were first prepared by the previously reported ethanol injection method with some modification [23,24]. Six formulations were established to develop and optimize luteolin-loaded chitosomes, as presented in Table 1. These included empty anionic liposomes (F1), varying luteolin loads (F2–F3), and different chitosan concentrations (F4–F6). Phosphatidylcholine (PC) (Lipoid S100), cholesterol, and phosphatidylserine in a 7.5:1.5:6.7, respectively, were dissolved in 1.5 mL absolute ethanol. The phospholipids dissolved in ethanol were added dropwise with a syringe to 5 mL of 0.1 M acetate buffer (pH 4.4) while stirring magnetically at 800 rpm for 1 h at room temperature 25 °C. The empty liposomal suspension (F1) was stored at 4 °C overnight for stabilization. For the LUT loading step, different luteolin concentrations were evaluated (20 mg% (*w*/*v*) (F2) and 40 mg% (F3) (*w*/*v*)) by dissolving in the ethanolic solution of the phospholipids before the addition to the aqueous medium [25]. The chitosan coating was prepared by titration method as follows: 100 mg% (*w*/*v*) chitosan solution was prepared by dissolving in 0.1 M acetate buffer (pH 4.4) under vigorous stirring overnight [26]. Varying concentrations were added dropwise to a constant volume of liposomes with moderate magnetic stirring (500 rpm) at room temperature (F4, F5, and F6). Stirring was maintained at room temperature 25 °C for 1 h, and the formulations were stored overnight in a refrigerator to equilibrate [27].

### 2.2. Physicochemical Characterization of CS-NPs

#### 2.2.1. Particle size, Zetapotential, and Polydispersity Index Measurement

Particle size (PS) and ζ-potential (ZP) analysis were determined using photon correlation spectroscopic technique using Malvern Zetasizer ZS using a dynamic light-scattering particle size analyzer (Malvern Instruments Ltd., Malvern, UK). Vesicle dispersions were diluted with water to an appropriate scattering intensity at 25 °C and at an angle of 173° and sonicated for 5–10 min prior to examination. For particle size analysis, samples were placed in square glass cuvettes, while those for ZP were placed in ζ-cells. Measurements were done in triplicate, and the values were recorded as the mean ± standard error of the mean (SD).

#### 2.2.2. Determination of Entrapment Efficiency (EE%)

Ultrafiltration technique was used in determining entrapment efficiency (EE) of LUT using Centrisart^®^ (MWCO 100,000; Sartorius, CA , USA) [28]. Luteolin-chitosomes (2.5 mL) were placed in the outer tube then centrifuged for 15 min at 4000 rpm. After centrifugation, the free unentrapped LUT was collected from the inner tube and measured spectrophotometrically at λmax 350 nm (Shimadzu UV spectrophotometer, 2401/PC; Shimadzu Corporation, Kyoto, Japan). All measurements were done in triplicates and %EE was calculated according to the following equation (Equation (1)) [4,29]:

(1) $$\mathrm{EE}\%=\frac{\left(\mathrm{Total}\;\mathrm{drug}-\mathrm{Free}\;\mathrm{drug}\right)}{\mathrm{Total}\;\mathrm{drug}}*100$$

#### 2.2.3. Transmission Electron Microscopy

The morphology of the prepared blank or drug-loaded NPs was determined by transmission electron microscopy (TEM). A drop of sample was placed on a carbon coated grid and left for 1 min to allow adherence on the carbon substrate. Following that, samples were stained with a saturated solution of uranyl acetate for 30 s preceding the microscopical examination [22].

#### 2.2.4. In Vitro Release Study

In vitro release studies were conducted using dialysis bags (ViskingR 36/32, 28 mm, MWCO 12,000–14,000; Serva, Heidelberg, Germany) [30]. LUT was accurately weighed to obtain a concentration equivalent to 0.2 mg/mL. One milliliter of the preparations (LUT suspension, LUT- loaded anionic liposomes, and LUT-CHS) was added to dialysis bags. The release medium (50 mL) consisting of PBS (pH 6.4) containing 0.5% Tween-80 was stirred continuously at 100 rpm at 37 °C [31,32,33,34]. Following that, one milliliter of the release medium was withdrawn at fixed time intervals (0.25, 0.5, 1, 3, 6, 10, and 24 h) and replaced with fresh medium to attain sink conditions. LUT concentration was measured spectrophotometrically at 350 nm in triplicate [5].

#### 2.2.5. Release Kinetics of Chitosan Nanoparticles

Release profiles from the in vitro release studies were also examined for release kinetics using the DDSolver software program. Six popular and important criteria were determined such as: adjusted coefficient of determination (Rsqr_adj), Mean standard Error (MSE), n (release exponent), Model Selection Criterion (MSC), and β (an indicator of the mechanism of transport of a drug through the polymer matrix). The highest MSC and R^2^ adjusted values were used for evaluating Higuchi, Hixson-Crowell, Korsmeyer-Peppas, and Weibull models [35].

#### 2.2.6. Stability Study

Optimized selected LUT-CHS formulation was stored in amber glass bottles at 4 °C and stability was inspected over a period of 6 months. Samples were investigated for PS, PDI, ZP, and entrapment efficiency [36]. Besides, visual examinations were carried out regularly to detect any signs of physical instability, such as precipitation, aggregations, or separation.

#### 2.2.7. In Vitro Mucoadhesion Test

Mucoadhesion testing was assessed by the mucin-particle method through measuring the changes in ZP [21,37]. Equal volumes (5 mL) of both the optimized NPs dispersion and the freshly prepared mucin solution (1%) were mixed and vortexed for 1 min. Following that, the ZP of the mucin solution, NPs dispersion, and the mixture was determined using zetasizer. Results were the mean values of three runs.

### 2.3. In Vivo Studies

#### 2.3.1. Animals

Animal experiments were performed according to approval and ethical guidelines of Animal Care and Use Committee of Cairo University (CU-IACUC) (Permit Number: CU-II-F-4-22) which complies with the Guide for the Care and Use of Laboratory Animals published by the US National Institutes of Health (NIH Publication No. 85-23, revised 2011). Adult Swiss Albino male mice (18–22 g) were collected from the animal house of the National Research Center, Cairo, Egypt. Animals were allowed to adapt for at least 1 week before the experiment started. Four to five mice per cage were kept under humidity and temperature controlled conditions with a 12-h light/dark cycle and free access to food and water. All efforts were made to minimize animal discomfort and suffering.

#### 2.3.2. Induction of Sporadic Alzheimer’s Disease (SAD)

Intracerebroventricular (ICV) injection of STZ, as first described by Pelleymounter et al. and modified by Warnock, was performed in this study for cerebral vein penetration avoidance [23,24,25]. Mice were anesthetized with i.p. thiopental (50 mg/kg) [38], and the head of the mouse was stabilized using downward pressure above the ears. The needle was inserted directly through the skin and skull into the lateral ventricle, which was targeted by visualizing an equilateral triangle between the eyes and the center of the skull to locate the bregma. This allowed the needle to be inserted at the following coordinates from the bregma: 1 mm mediolateral, −0.1 mm anteroposterior, and −3 mm dorsoventral. The mice behaved normally approximately 1 min following the injection.

#### 2.3.3. Experimental Design

**Figure 1:**
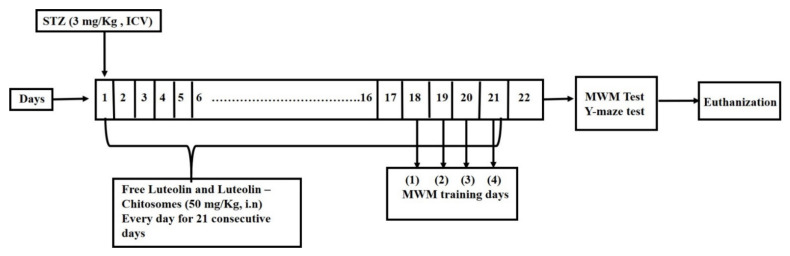
A schematic representation for the experimental design.

Mahagan HS et al. and Elkarray S M et al. [39,40] reported that the dose to be administered nasally is often 2 to 10 times lower than the oral doses. Therefore, the nasal LUT dose is 5 mg/kg which is equivalent to 50 mg/kg LUT orally. The mice were randomly divided into four groups (8 mice/group). Group I: mice received an ICV injection of saline once and an intranasal saline injection for 21 successive days which served as a normal control group. Group II: mice received streptozotocin (STZ) (3 mg/kg, ICV) once which served as an SAD model. Group III: mice received STZ (3 mg/kg, ICV) followed by intranasal administration of 100 μL in each nostril (0.02 mg LUT) luteolin suspension in saline (2 mg/kg/day) after 5 h and then every day for 21 successive days [4,41]. Finally, the same protocol was applied to Group IV in which the mice received STZ (3 mg/kg, ICV), followed by 100 μL luteolin-chitosomes (0.02 mg LUT) equivalent to (2 mg/kg/day) administered intranasally after 5 h and then every day for 21 consecutive days [3,4] as shown in Figure 1.

#### 2.3.4. Intranasal Administration

Intranasal formulations were administered by dropping a single daily dose of the formulation until the 21st day using a tube made of polyethylene and attached to a micropipette which was inserted approximately 3 mm into nostril of the mouse without anesthesia. The volume of the intranasal administration was 100 μL, and the drug was administered into the right and left nostrils. During and for 20 s after the nasal drop, animals were maintained in a supine position to allow the drug to reach the olfactory region or the upper part of the nasal cavity where it would have direct access to the brain [10]. Each experiment was carefully performed at the same period of the day to avoid deviations in animal’s behavior [4].

#### 2.3.5. Behavioral Assessment

##### Y-Maze

The spontaneous alternation behavior in the Y-maze task was used as a measure to evaluate short-term memory [42]. The apparatus used in the current study consisted of three metallic arms that formed a Y-shaped maze. Each arm was 35 cm long, 10 cm wide, and 25 cm high in dimensions and extended 120° from the center of the platform. Typically, normal mice prefer to explore the new arm of the maze rather than a previously used one. The investigation was then performed on two successive days. On the 1st day of training, each mouse was placed at the platform center and allowed to move freely through the maze for 8 min. On the test day, during the 8-min session, the sequence of arms each mouse entered was recorded. The maze was cleaned by ethanol (70%) after every mouse and also during each session, to remove any olfactory cues, and hence, reduce any errors in the observations. An actual alternation was determined as consecutive entries into all three arms, known as overlapping triplet sets. The possible alternations were determined as the total number of entries to arm. The percentage of spontaneous alternation behavior was then calculated using the following equation [41]:

(2) $$\%\;\mathrm{spontaneous}\;\mathrm{alternation}\;=\frac{\mathrm{Actual}\;\mathrm{alternations}}{\mathrm{Total}\;\mathrm{number}\;\mathrm{of}\;\mathrm{possible}\;\mathrm{alternations}\;}*100$$

##### Morris Water Maze (MWM)

The Morris water maze (MWM) measures the visuospatial memory and the learning capability of animals [43]. The apparatus used in the current test consisted of a large circular stainless-steel pool that was half-filled with water adjusted at room temperature 25 °C with dimensions of 150 cm in diameter and 60 cm in height [41]. Two threads were placed perpendicular to one another, dividing the pool into four quadrants. A submerged black platform with dimensions of 10 cm in width and 28 cm in height was placed 2 cm below the water surface inside the targeted quadrant of the pool. The platform location was kept persistent during the test. In order to make the platform invisible, the water was rendered opaque by adding non-toxic purple colored dye. It was supposed that normal animals would learn to quickly swim towards the platform directly, and hence consume a shorter time. The procedure was carried out on five consecutive days [44]. On the first 4 days of the test, each mouse was trained to escape and climb onto the hidden platform (2 consecutive trials per day × 4 days), with a gap interval of 15 min at least between the trials. For each trial, the maximum time allowed was set to be 60 s. If the mouse was able to locate the hidden platform throughout the selected 60 s, it further remained there for extra 20 s before being removed. However, if the mouse failed to find the hidden platform during the designated time, it was gently directed onto the platform and allowed to remain there for 20 s. Then, the mean escape latency (MEL) time was determined as the required time for each mouse to locate the hidden platform. It was determined during each of the trials over the four days of the test. In addition, it was measured as an acquisition or learning index [45]. On the fifth day, the mice were undergoing a session of probe-trial where the platform was removed from the pool, then each mouse was allowed for 60 s to explore the pool. The index of retrieval or memory was finally recorded as the time that each mouse spent in the target quadrant, where the hidden platform was formerly located [46].

#### 2.3.6. Histopathology

Brain tissues from mice in different groups were collected and fixed using 10% formalin. Paraffin-embedded tissue blocks were prepared and divided into sections on a glass slide with a microtome. Then, the paraffin sections were routinely stained with HE [47] and examined using a light microscope linked to a digital camera (Olympus BX43 light microscope-Olympus DP27 digital camera). Other brain sections were prepared for Nissl and Congo red stains.

#### 2.3.7. Immunohistochemistry

Both tissue preparation and immunohistochemical examination were carried out. Briefly, after the rehydration process, epitope retrieval was performed with heat. The tissue sections were blocked with bovine serum albumin and hydrogen peroxide. A primary anti-mouse antibody against glial fibrillary acidic protein (GFAP) (sc-166458, Santa Cruz Biotechnology, Inc., Heidelberg, Germany, dilution of 1:200) was incubated overnight in a humidified chamber at 4 °C. After a washing step to remove excess primary antibody, a secondary HRP-labeled antibody was applied (ab97023- Goat anti-mouse HRP-labeled antibody, Abcam, Cambridge, UK, 1:200) and incubated at room temperature 25 °C for 2 h. After washing, 3,3′-Diaminobenzidine (DAB) Substrate (Pierce™ DAB Substrate Kit (34002), Thermo Scientific, Inc., Rockford, IL, USA) was used to develop the reaction, which was examined by light microscopy. Negative control slides were prepared by removing the primary antibody incubation step. Images were captured with cell Sens Dimension software, Olympus, Tokyo, Japan, to measure the area occupied by GFAP-positive cells.

#### 2.3.8. Measurement of Biochemical Parameters

For determination of various levels of biochemical markers, the brain homogenized in ice-cold physiological saline (10% *w*/*v*). Homogenization was carried out using a Potter-Elvehjem tissue grinder (Thermo Scientific Waltham, MA, USA) at a speed of 14,000 rpm for 20 s. Finally, the supernatants were stored at −80 °C in an Isotemp freezer (Basic Thermo Fisher Scientific Waltham, MA, USA) until further analysis [48,49,50].

##### Estimation of Oxidative Stress Markers (MDA, GSH, and NRF2)

Lipid peroxidation was assessed by measuring malondialdehyde (MDA) levels according to the method described by Janero [51]. The content of brain glutathione (GSH) was spectrophotometrically determined using Ellman’s reagent. The results are expressed as mol/mg protein [52]. Nrf2 was quantified using the ELISA kit (MyBioSource, Inc., San Diego, CA, USA) according to the manufacturer’s instructions. The results are expressed as ng/g tissue [52].

##### Estimation of Pro-Inflammatory Mediators (NOS, COX-2, NF-Κβ, and TNF-α)

COX-2, NF-κB p65, and TNF-α were measured using ELISA kits purchased from COSABIO, Inc. (Houston, TX, USA), R&D Systems Inc. (Minneapolis, MN, USA), and RayBiotech Inc. (Norcross, GA, USA), respectively. The procedures were performed according to the manufacturers’ guidelines. The results are presented as pg/g tissue for TNF-α and ng/g tissue for COX-2 and NF-κB p65 [53]. The Griess assay was used to determine the total levels of NO in the brain tissues. The results are presented as ng/g tissue.

##### Determination of Mouse MMP-9(Matrix Metalloproteinase 9)

MMP-9 was quantified using ELISA kits (Wuhan Fine Biotech Co., Ltd., Wuhan, China). Procedures were carried out according to the manufacturer’s guidelines. Results are presented as ng/g tissue.

##### Estimation of Amyloidogenesis and Tauopathy (*Aβ1-42* and Tau)

*Aβ1-42* and tau were measured using mouse ELISA kits purchased from Novus. The procedures were performed according to the manufacturers’ instructions. Results are presented as ng/g tissue for tau and pg/g tissue for *Aβ1-42* [53].

##### Estimation of The Mouse cAMP Response Element Binding Protein (CREB) Transcription Factors

CREB was quantified using ELISA kits (COSABIO, Inc., Houston, TX, USA). The procedures were carried out according to the manufacturer’s instructions. The results are expressed as pmol/g tissue [54].

#### 2.3.9. Safety Studies

An observational nasal irritation test was performed. During nasal administration, the effect of the administered formulations including LUT suspension, LUT loaded chitosomes, LUT loaded liposomes and blank chitosomes on the nasal mucosa was investigated by visual observation. Upon intranasal administration, the behavior of the animals was carefully monitored, and the number of animals showing signs of mucosal inflammation was ascertained. Signs of mucosal inflammation encompassed sneezing, itching, and discomfort. The nasal mucosa irritation index reported by El naggar et al. [4] that entailed four degrees of irritations according to the percentage of animals showing irritation signs was applied. These include strong irritation (more than 60% of the animals), moderate irritation (from 30% to 60%), mild irritation (from 10% to 30%), and no irritation (up to 10%).

#### 2.3.10. Statistical Analysis

Statistical analysis was performed using the software (GraphPad Prism software, version 8.01, Inc., San Diego, CA, USA). The results are expressed as the mean ± SD. For the Morris water maze test, escape latency was analyzed using two-way repeated measures ANOVA. The data that were not included in repeated measures were analyzed with one-way ANOVA followed by the Tukey post-hoc test for multiple comparisons. Differences were considered significant at a *p* value < 0.05.

## 3. Results and Discussion

### 3.1. Preparation and Characterization of Chitosomes (CHS)

Phosphatidylserine is a negatively charged synthetic phospholipid which imparts a negative surface charge on the empty anionic liposomes to reach a value of −24.9 ± 4.21 mV. Consequently, the presence of a negative charge on the liposomal surface confirms the formation of a stable homogenous preparation (Polydispersity index (PDI) = 0.3014 ± 0.06) and optimum coating with positively charged chitosan. Loading LUT (20 and 40 mg%) caused a significant increase in the vesicular size of LUT-loaded liposomes (212.3 ± 2.18 nm and 320.0 ± 4.23 nm, respectively) compared with the unloaded formulation (184.6 ± 1.85 nm). This increase may indicate the existence of hydrophobic LUT in the outer phospholipid layer of anionic liposomes compared with an aqueous core. With respect to the EE%, different LUT concentrations were loaded into anionic liposomes, and the results (Table 2) revealed that doubling the dose from 20 to 40 mg% did not double the EE%. However, Lip-LUT_20_ exhibited higher luteolin entrapment (80.6% ± 1.28%) compared with Lip-LUT_40_ (77.9% ± 1.33%). This may be attributed to the limited amount of luteolin that can be entrapped in the phospholipid bilayer of anionic liposomes. Therefore, 20 mg% of luteolin loading was used for subsequent chitosomal preparations.

With respect to the chitosan decorated nanoparticles (chitosomes, CHS), an initial screening of the optimal concentration of chitosan solution was conducted as shown in Table 2. The results indicated that concentration of 4 mg%, (LUT-CHS_4%,_ F5) yielded the highest quality attributes (PS 412.8 ± 3.28 nm, PDI 0.378 ± 0.07, ZP 37.4 ± 2.13 mv, and EE% 86.6 ± 2.05%) (Supplementary Figure S2) compared with 2 mg% (LUT-CHS_2%,_ F4), and 8 mg% (LUT-CHS_8%,_ F6). The results showed that different chitosan coatings generated larger particle sized liposomes. Furthermore, chitosan coating led to a significant inversion to positive ZP from negative ZP because of the cationic nature of chitosan. However, no significant increase was noted in the magnitude of the surface charge between LUT-CHS_4%_ (F5) and LUT-CHS_8%_ (F6) despite doubling the chitosan concentration. This “plateau effect” in ZP may be attributed to the saturation of the liposomal surface with chitosan, which is consistent with previously reported data [22]. Of note, the significant increase in EE% of LUT-CHS_4%_ (86.6% ± 2.05%) compared with Lip-LUT_20_ (80.6% ± 1.28%) may result from the incorporation of more LUT inside the chitosan matrix beside the phospholipid bilayer of the anionic liposomes.

Upon storage over a period of 6 months, the selected LUT-CHS preparation showed good physical stability without any signs of aggregation, separation or drug precipitation at 4 °C (Table 3). Regarding the quality attributes, a non-significant increase was observed in PS which may be due to the slight swelling of the chitosan polymer matrix. Such swelling may lead to degradation of the chitosan matrix which is reflected by the decrease in the ZP after 6 months (34.3 ± 3.04 mV) compared to the initial point (37.4 ± 2.13 mV). Consequently, EE% decreased after 6 months (82.8 ± 1.15%) compared to the first EE% (86.6 ± 2.05%) due to the leakage of the entrapped LUT. The obtained results were in a good agreement with those reported by Delan W et al. [36]. Therefore, the authors recommend further stability improvement via lyophilization.

### 3.2. Transmission Electron Microscopy (TEM)

**Figure 2:**
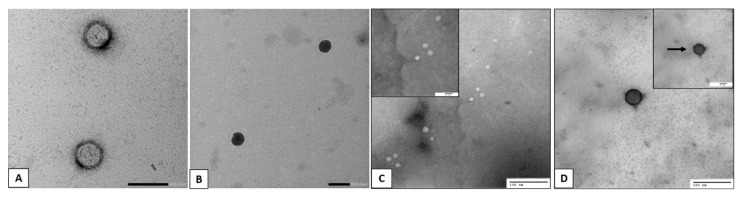
TEM of (**A**) empty Liposomes (206,000× magnification), (**B**) luteolin-loaded liposomes (183,000× magnification), (**C**) empty CHS (120,000× magnification) and (**D**) luteolin-loaded CHS (150,000× magnification). Arrows point to the chitosan coating layer.

Representative micrographs of the empty liposomes, Lip-LUT, empty CHS and LUT-CHS particles (Figure 2a–d) showed that both samples possessed a characteristic spherical vesicular system with an evident core and phospholipid bilayer membrane shell. Furthermore, LUT-CHS and Lip-LUT (Figure 2b,d) exhibited a denser core compared with that of the empty Lip and empty CHS (Figure 2a,c). Our TEM micrographs further confirmed the existence of a dark chitosan sheath over the vesicular surface in addition to the absence of aggregations or undesirable structures. It is worth mentioning that the size of the vesicles measured by TEM was noticeably smaller than the average particle size determined by the zetasizer. The reason behind this is that the presence of hydrodynamic layers around the vesicles may lead to an overestimation of the PS measurements when determined by the zetasizer [55].

Moreover, the principles comprised in the analysis in both techniques are different. Regarding dynamic light scattering (DLS), it is an intensity-based technique where the resultant size distribution is the average hydrodynamic size of the nanoparticles and it is usually affected by the presence of large particles, dust, or aggregates [56], while the microscopic analysis by TEM is mainly based on nanoparticle tracking analysis (NTA) and the finding is almost done after the routine procedure of air drying of a nanoparticles-containing droplet on the TEM grid. Such a technique (NTA) is a number-based technique that tracks individual nanoparticles (single-particle tracking) [56]. Consequently, the latter can provide accurate number-based average dimensions with lowest bias for samples free from artefacts [57].

### 3.3. In Vitro Release Study and Release Kinetics

**Figure 3:**
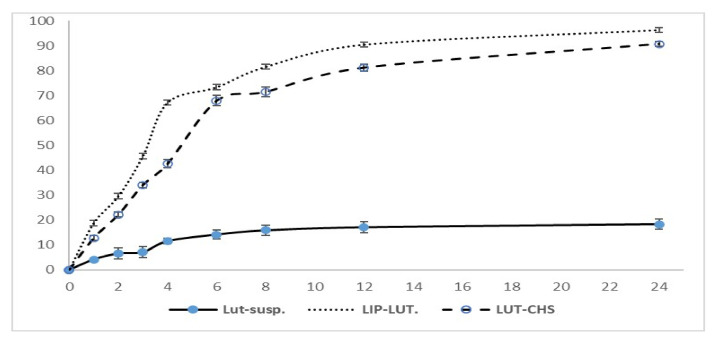
In vitro release profile of luteolin in PBS (pH 6.4) containing 0.5% Tween-80 from LUT suspension, LUT-loaded liposomes and LUT loaded chitosomes. Results represented as mean ± SD, n = 3.

The rationale of selecting release medium consisting of PBS (pH 6.4) was to mimic the nasal environment because the nasal mucosal pH is approximately 5.5–6.5 [58]. On the other hand, LUT is classified in the Biopharmaceutical Classification System (BCS) as a Class II drug which is characterized by low solubility in aqueous media [5,59]. Consequently, in order to achieve the sink conditions, several studies reported the addition of 0.5% tween 80 in the release medium to maintain the best condition for LUT dissolution [60,61,62]. In the current study, all examined formulations offered a significantly higher release profile in comparison to LUT suspension. When LUT was incorporated in anionic liposomes, it showed a significant enhancement in release compared with the LUT suspension. This might be attributed to the nano-solubilization of the poorly soluble LUT and the drug being in a molecular state [5]. The cumulative percentage release of LUT from Lip-LUT and LUT-CHS was over 90% over a period of 24 h compared with 18% for the LUT suspension. In addition, the release pattern of LUT from CHS was similar to that of liposomes with a lower retardation. As shown in Figure 3, the percentage of drug released over a period of 2 h was only 22% from LUT-CHS compared with 30% from Lip-LUT. These results indicate the amenability of chitosomes to control the release of loaded LUT. This may be ascribed to the different release mechanisms incorporated for Lip-LUT and LUT-CHS. Regarding Lip-LUT, the delayed release of LUT may be attributed to the time required for drug partitioning from the liposomes to the aqueous medium, which is considered a predominate step in release rather than dissolution. In contrast, LUT-CHS exhibited a more prolonged release effect because of the proper LUT encapsulation in the chitosan matrix in addition to its incorporation into the phospholipid bilayer. Consequently, the drug release mechanism from CHS is believed to occur by both degradation of polymer and diffusion from the liposomes.

The release mechanism of LUT from CHS was further confirmed by release kinetics modeling. Depending on the R^2^-adjusted and MSC values (Table 4), the Weibull model was found to be the best fitting model compared with the others. It was reported that the value of the exponent “β” is a parameter of the drug transport mechanism through the polymeric chitosan matrix [63]. The assessed values of β ≤ 0.75 suggests that the diffusion occurred according to Fick’s law, whereas “β” values between 0.75 and 1 suggest that a combined mechanism of both Fickian diffusion and swelling controlled release should be considered [35,64]. In the current investigation, the “β” value was 0.900, indicating that the complex release mechanism was the predominant mechanism of LUT release. This mechanism indicates the nature of chitosan as a swellable polymeric material that undergoes degradation via a hydrolytic process [65]. Therefore, the water channels formed in the chitosan matrix allow LUT diffusion and assist in the degradation of chitosan itself.

### 3.4. In Vitro Mucoadhesion Test

Mucin-particle method was used to assess the mucoadhesive properties of the developed chitosomes by determining the changes in ZP. A significant change in surface properties was observed for formulas coated with chitosan (F4, F5, and F6) after being mixed with mucin solution at pH 6.5; where the ZP of F4, F5 and F6 was obviously decreased and shifted from + 28 ± 2.94, +37.4 ± 2.13, and +36.2 ± 3.04 to −6.42 ± 0.13, −5.44 ± 0.22, and −5.67 ± 0.15 respectively. Interaction between the sialic groups of the negatively charged mucin and the surface layer of the (positively charged) chitosan coat on the chitosomes was anticipated to decrease the ZP. The observed results indicate the high affinity of chitosan to mucin particles and highlight the interaction between chitosan and mucin particles [21,37]. Chitosan’s mucoadhesive effect is based on the electrostatic interactions with the negatively charged endothelial surface of mucous membranes. The developed chitosomes then absorb water from the surrounding mucus and swell leading to the formation of a gel-like structure. Consequently, chitosan coated-NP will remain in the nasal cavity for prolonged periods of time surmounting the mucociliary clearance and leading to an improved drug absorption from nasal mucosa [21,66].

### 3.5. In Vivo Study

Streptozotocin (STZ) is a methyl nitrosourea originally developed as an anticancer agent which was found to induce diabetes in animal models of insulin-dependent (type 1) diabetes after systemic administration [67]. However, after intracerebroventricular (icv) administration at a subdiabetogenic dose, it decreases brain glucose uptake and triggers pathological and neurobehavioral features such as that of AD [41,67,68]. Therefore, the ICV-STZ mouse model was applied to this current study as an animal model for AD.

#### 3.5.1. Behavioral Test

##### Y-Maze Test

**Figure 4:**
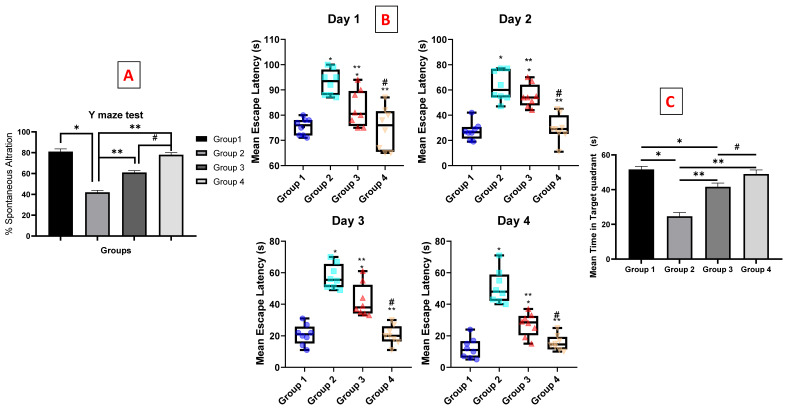
Behavioral tests on effect of luteolin-chitosomes on ICV-STZ mouse model. Animals were divided into 4 groups, eight animals in each group (n = 8), the first one was the normal control group. The second group was the positive control group that received STZ (3 mg/kg, ICV). The last two groups were all ICV-injected, first with STZ (3 mg/kg) followed by intranasal administration of luteolin suspension (50 mg/kg, every day for 21 days) and luteolin-loaded chitosomes (50 mg/kg, i.n. for 21 days) respectively. (**A**) Y-maze test that measured the percentage of spontaneous alternation, (**B**) box and whisker plots of mean escape latency (MEL), and (**C**) mean time in target quadrant. Statistical analyses for the Morris water maze test (escape latency) were analyzed using two-way repeated measures ANOVA. The data that were not included in repeated measures were analyzed with one-way ANOVA followed by the Tukey post-hoc test for multiple comparisons. Each value was expressed as mean ± SD. *: Statistically significant different from the normal group at *p* < 0.05, **: Statistically significant different from the positive control group (STZ, 3 mg/kg) at *p* < 0.05. #: Statistically significant different from the luteolin suspension group (50 mg/kg).

The Y-maze is used to determine the willingness of rodents to explore new environments [69]. The percentage alternation was significantly decreased by about two folds in group 2 (STZ, 3 mg/kg) compared with normal mice receiving saline (group 1). These findings confirmed the fact that the STZ administration led to a decline in spatial and working memory as reported by Fronza et al. [70]. The administration of both luteolin suspension and chitosomes (50 mg/kg, i.n) significantly improved the acquisition of short-term memory by approximately 45.23% and 85.7%, respectively, compared with the STZ group. These results are consistent with the previously reported study showing improvements in spatial recognition memory in the Y-maze test after luteolin consumption [71]. Moreover, a significant improvement was observed in the LUT-loaded CHS group (group 4) compared with the luteolin suspension group (group 3), as shown in Figure 4A. These results indicate the presence of an additive effect of the nano-chitosomes on LUT activity. This beneficial effect may be due to the nanosolubilization of the poorly soluble luteolin, which in turn, improves drug biopharmaceutics properties. Additionally, it may result from the mucoadhesive property of the chitosan coat which improves the nasal residence time and, hence, increases brain uptake [72].

##### Morris Water Maze (MWM) and Mean Escape Latency (MEL)

The MWM is a meaningful behavioral test indicating hippocampal-dependent spatial learning and long-term spatial memory in rodents [73,74]. It is a familiar test for assessing spatial navigation and learning capabilities. Behavioral parameters such as thigmotaxis suppression and recognition of the hidden platform as an escape are critical factors in the MWM performance. These deficits can badly affect searching behavior and limit the acquisition of navigation that is required to solve the task effectively. Generally, the behavior of thigmotaxis dominates as the animals look for wall contact where they presumably feel safer [75]. However, non-spatial pre-training (NSPT) provides exposure to learned task components, important for navigation and non-spatial search strategy development. Therefore, NSPT can depress thigmotaxis and facilitate the acquisition of behaviors linked to successful task completion [76]. In the current study, training of each mouse (NSPT) was done on the first 4 days of the test where each mouse was subjected to two successive trials with an interval gap of 15 min at least between the trials, therefore, the difficulty of thigmotaxis was overcome.

On the first day, all mice recorded comparable MEL values with no significant differences in time to reach the platform. In subsequent days, mice assigned to the “LUT-SUSP” (group 3) exhibited increased efficiency in locating the platform, resulting in a significantly lesser MEL compared with that measured for the STZ control (group 2). A difference of 24.4, 21, and 15.8 s were recorded on the second, third, and fourth days, respectively. These data are in agreement with a previously reported study which demonstrated that mice fed with LUT had the ability to use the visual cues of the extra maze to resolve the acquisition task, indicating improved spatial learning and memory capability [74]. With respect to the nano-chitosomes (group 4), the mice displayed a significant improvement on cognitive impairment compared with the luteolin suspension (group 3), with no significant differences in MEL relative to group 1 throughout the testing period (Figure 4B). These findings emphasize the significant roles of nano-chitosomes in improving LUT solubility, absorption, brain uptake, and improved learning and memory function. In conclusion, LUT-CHS may significantly mitigate shortages in cognition and may be administered at doses much lower than the recommended therapeutic dose.

##### Morris Water Maze (MWM) and The Time Spent in The Target Quadrant

Results demonstrated that mice treated with 3 mg/kg ICV-STZ (group 2) showed a shortened average time spent in the target quadrant by almost half (0.47-fold reduction) relative to the normal group (group 1) (*p* *<* 0.05). The group receiving LUT suspension (group 3) (50 mg/kg i.n. once daily for 21 consecutive days) also spent a longer (by 1.7 fold) time in the target quadrant than that measured for the ICV-STZ group 2 (*p* *<* 0.005). Besides, group 3 demonstrated an 0.8-fold reduction in time compared to that spent by the normal group 1. On the other hand, there was a non-significant difference between the time measured for group 4 that received LUT-CHS (50 mg/kg, i.n) compared to the normal group, while the time spent by this group in the target quadrant was significantly longer: 24.33 s longer than that determined for the ICV-STZ group 2, as shown in Figure 4C. Finally, the nanochitosomes group (group 4) showed a statistically longer time spent in the target quadrant (1.2 fold) compared to LUT suspension (group 3).

#### 3.5.2. Histopathology

**Figure 5:**
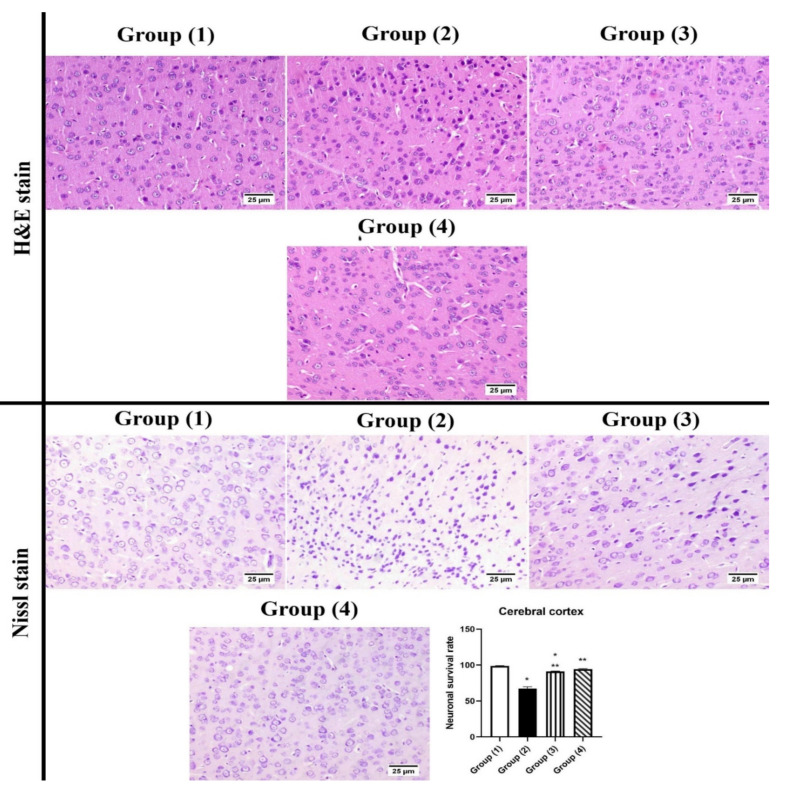
Photomicrograph of brain, cerebral cortex (H&E and Cresyl violet stains). Normal hippocampus in group (1), group (2) showed numerous shrunken neurons. Group (3) showed few degenerated neurons. Group (4) showed apparently normal cerebral cortex. The chart represents neuronal survival rate, statistical analyses were performed using ANOVA (one-way analysis of variance) followed by Tukey post hoc test. Each value was expressed as mean ± SD. *: Statistically significant different from the normal group at *p* < 0.05, **: Statistically significant different from the positive control group (STZ, 3 mg/kg) at *p* < 0.05.

The examination of the cerebral cortex of group (1) showed normal histological structure. Meanwhile, group (2) “STZ-ICH” revealed several scattered dark degenerated neurons that were associated with diffuse gliosis. Few shrunken neurons were detected in group (3) “LUT-SUSP”. The highest protection was noticed in group (4) “LUT-CHS” which showed an apparently normal histological structure of the cerebral cortex. The neuronal survival rate showed a significant decrease in group (2) compared to other experimental groups. However, the absence of significant difference was recorded between various treated groups (Figure 5).

**Figure 6:**
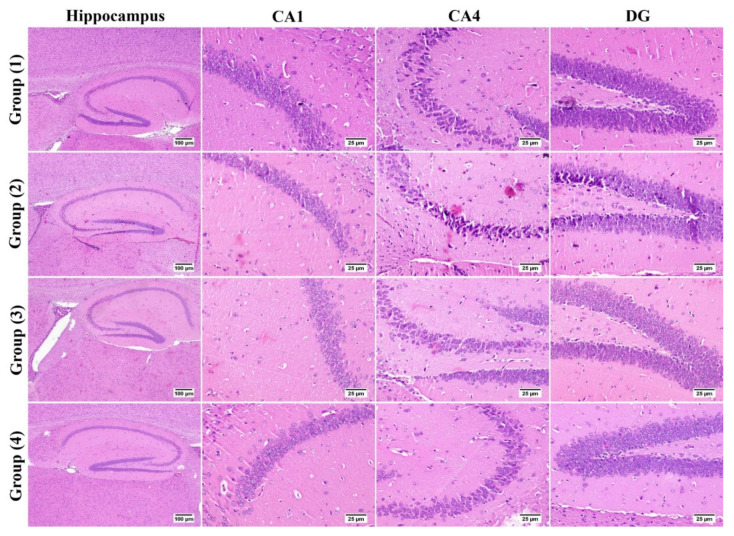
Photomicrograph of brain, hippocampus region (H&E stain). Normal hippocampus in group (1), group (2) showed marked degenerated neurons in CA4 and DG regions. Group (3) showed few degenerated neurons. Group (4) showed apparently normal hippocampus.

Microscopic examination of hippocampus from group (1) revealed normal histological structure of all anatomical regions; CA1, CA2, CA3, CA4, and DG showed normal appearing neurons. On the contrary, group (2) “STZ-ICV” showed marked neuronal damage especially in the CA1, CA4, and DG regions of the hippocampus; these regions contained numerous dark degenerating neurons admixed with gliosis. Regarding group (3) “LUT-SUSP”, mild improvement was noticed; CA1, CA4, and DG showed only few necrotic cells. The best protective action against hippocampus damage was noticed in group (4) “LUT-CHS” in which all examined sections were apparently normal (Figure 6).

**Figure 7:**
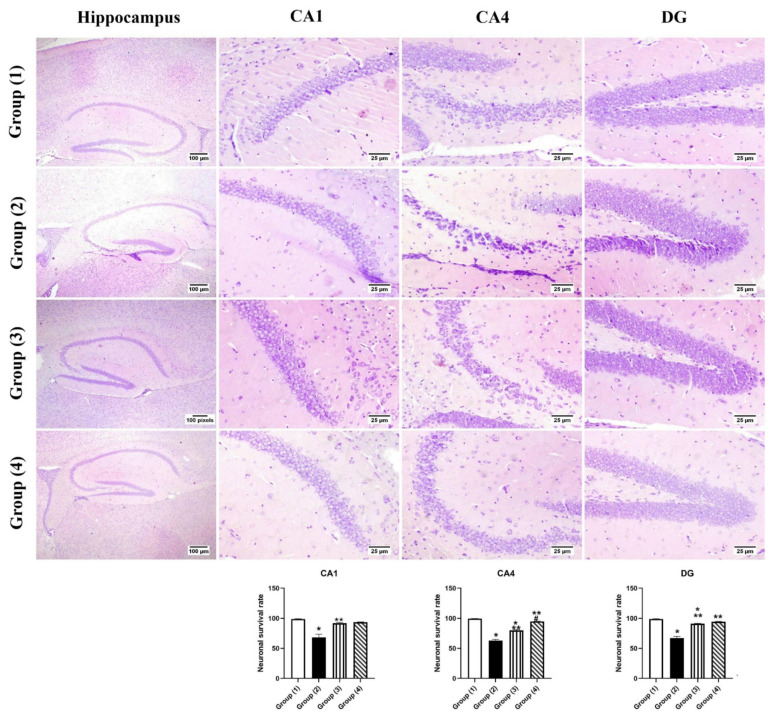
Photomicrograph of brain, hippocampus region (Cresyl violet stain), showing intact neurons within the different hippocampus regions in group (1), group (2) showed dark stained degenerated neurons within hippocampus regions, groups (3) and (4) showed apparently normal hippocampus. Charts represent neuronal survival rates, data expressed as means ± SE. Significant difference was considered at *p* < 0.05. *: Statistically significant different from the normal group at *p* < 0.05, **: Statistically significant different from the positive control group (STZ, 3 mg/kg) at *p* < 0.05. #: Statistically significant different from luteolin suspension group (50 mg/kg).

Regarding the neuronal survival rate within the hippocampus regions (Figure 7), survival neurons were significantly decreased in group (2) “STZ-ICV” compared to group (1) in CA1, CA4, and DG regions of the hippocampus. All treated groups exhibited a significant increase in the number of surviving neurons in all hippocampus regions. At the CA1 region, no significant statistical difference was observed between all treated groups. CA4 region showed an increased rate of neuronal survival especially in group (4) “LUT-CHS” which revealed a higher neuronal survival rate that was comparable to group (1).

**Figure 8:**
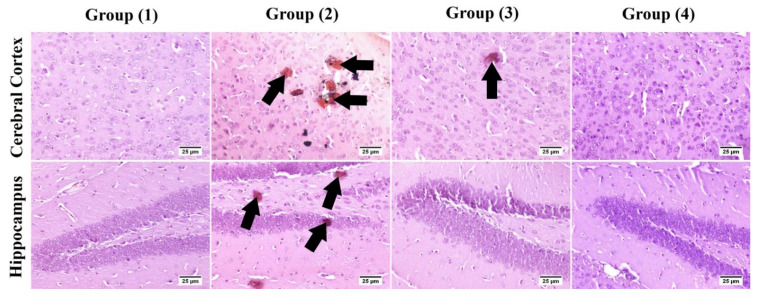
Photomicrograph of brain, Congo red for amyloid plaques visualization in the cerebral cortex and hippocampus. Group (2) showed marked increase in the number of amyloid lesions with few to absence in the treated groups (arrows).

*Congo red:* The number of amyloid plaques are illustrated in Table 5. Group (1) showed an absence of amyloid plaques in the examined brain sections. A significant increase in the number of amyloid depositions was detected in group (2) compared to other groups. A significant reduction was recoded in treated groups by 58% and 77.4% in groups (3) and (4) respectively when compared with group (2) (Figure 8).

#### 3.5.3. Immunohistochemistry

**Figure 9:**
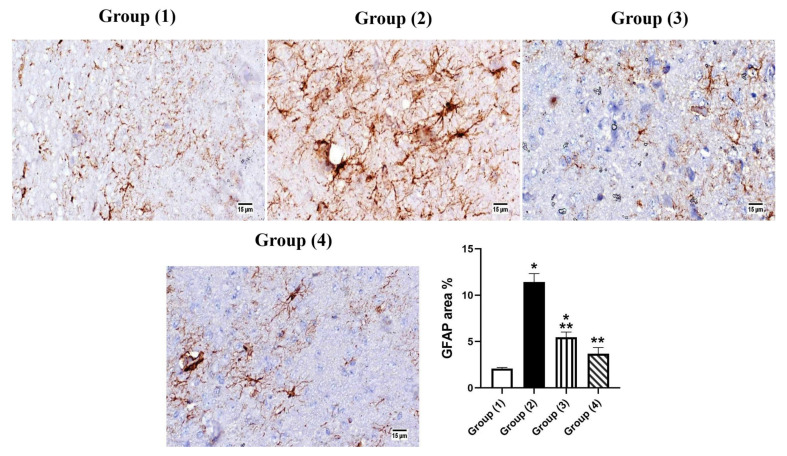
Photomicrograph immunohistochemistry of GFAP expression in brain tissue. Group (2) shows marked expression of GFAP; however, morphological difference between the two treated groups was not observed. The graph shows that the area % of GFAP was significantly elevated in group (2); values are expressed as means ± SE. Significant difference was considered at *p* < 0.05. *: Statistically significant difference from the normal group at *p* < 0.05, **: Statistically significant difference from the positive control group (STZ, 3 mg/kg) at *p* < 0.05.

The brain sections showed intense positive expression of GFAP in group (2), which showed a statically significant increase in area % expression compared to other groups. No significant difference was detected in all treated groups. The highest protection was recorded in group (4) “LUT-CHS” which revealed no significant variance in comparison with group (1) (Figure 9). The administration of STZ resulted in histopathological changes to the brain similar to those generated by AD [77]. Likewise, STZ-ICV model has been considered as a suitable model for Alzheimer’s (Singh and Kumar 2016). The detected histological lesions in the hippocampus prove the role of AD in changing memory and spatial learning potentials [78]. Nissl stain of the cerebral cortex showed marked reduction in the number of surviving neurons in the STZ administrated group, this neuronal degeneration resulted in synaptic dysfunction of the brain regions responsible for cognitive functions, with further memory disorders [79]. Previous studies have confirmed the relation of neuronal degeneration with memory impairment [80,81].

Our histopathological findings revealed the formation of Aβ plaques and neuronal degeneration in both hippocampus and cerebral cortex which was consistent with the findings of [78] in AD model. GFAP affects mitotic activity, astrocyte–neuron interaction, communication between cells, and repair of CNS injury [82]. Prominent astrogliosis demonstrated in GFAP immune staining of brain segments, mainly around the amyloid plaques, emphasized the role of astrocytes in the degradation of amyloid plaques via the astrocytic processes [83,84].

#### 3.5.4. Biochemical Parameters

##### Estimation of Oxidative Stress Markers (MDA, GSH and NRF2)

**Figure 10:**
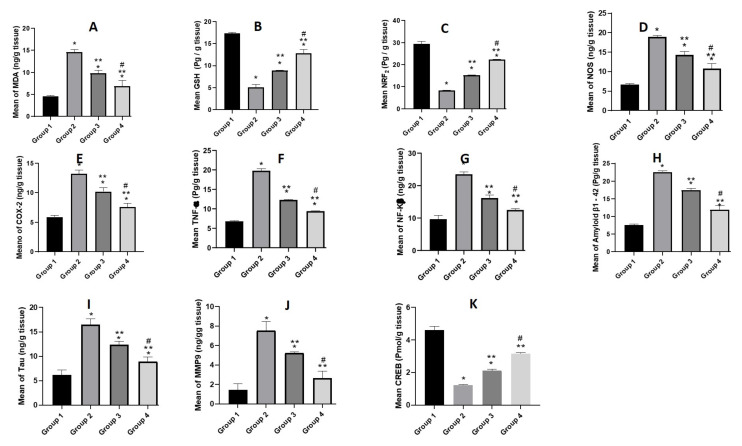
Biochemical assay where animals under investigation were divided into 4 groups; the first one was the normal control group. The second group was the positive control model group that received STZ (3 mg/kg, ICV). The last two groups were all ICV-injected first with STZ (3 mg/kg) followed by i.n. injection with luteolin suspension (50 mg/kg, for 21 days), and luteolin loaded chitosomes (50 mg/kg, i.n. for 21 days), respectively. The brains of the animals (n = 8) in each group were homogenized then centrifuged, and supernatants were used in the oxidative stress parameters assay of (**A**) mean MDA concentration, (**B**) GSH level and **(C)** NRF2 level. The proinflammatory mediators’ assay of (**D**) mean NOS level, (**E**) COX-2 level (**F**) TNF-α level, and (**G**) NF-Kβ levels. The amyloidogenicity and tauopathy of (**H**) mean of Amyloid β_1-42_ level, and (**I**) Tau level. Finally, (**J**) MMP9, and (**K**) CREB level. Statistical analyses were performed using ANOVA followed by the Tukey post hoc test, whereby each value was expressed as mean ± SD. * Statistically significant difference from the normal group (*p* < 0.05); ** statistically significant difference from the STZ model group, (*p* < 0.05); # statistically significant difference from the LUT susp group 50 mg/kg (*p* < 0.05).

The administration of STZ significantly increased the MDA and decreased the GSH levels in the brain tissue as shown in Figure 10A,B. These findings were supported by a previous study that showed treatment of normal mice with STZ (3 mg/kg, ICV) led to elevation of the brain MDA while reducing the glutathione levels (GSH) [71,85]. On the other hand, the administration of LUT-SUSP and LUT-CHS significantly mitigated the STZ-induced increase of lipid peroxidation. Results demonstrated that free LUT and LUT-CHS showed a 0.6- and 0.5-fold decrease in the MDA levels respectively compared to the STZ group. Regarding the GSH levels, both free LUT and LUT-CHS demonstrated 2- and 2.5-fold elevation in the GSH respectively. This may be associated with the increase of intracellular antioxidants and hence decrease of lipid peroxidation [74]. Such findings were in harmony with previously reported studies that related the underlying neuroprotective mechanism of luteolin (50 mg/kg) for 8 weeks to its antioxidant activity in a streptozotocin model [7,71].

The (Nrf_2_) is the nuclear factor erythroid 2-related factor 2; it is a vital redox-regulated gene that has an important role in combating oxidative stress. Furthermore, the level of Nrf_2_ in the nuclei declines in neurological disorders such as AD [86]. Consistently, in our AD model, the STZ group showed significant decrease in Nrf_2_ by 3.5 folds compared to normal group. Upon administration of free LUT and LUT-CHS, 2- and 2.6-fold elevation was observed respectively compared to the STZ group as shown in Figure 10C. This effect reflects the important role of flavonoid LUT in modulating NRF_2_ level. These results came in agreement with our previous antioxidative stress results where it was confirmed that LUT treatment reduced the ROS via the activation of Nrf2 [87].

##### Estimation of Pro-Inflammatory Mediators (NOS, COX-2, NF-κβ, and TNF-α)

A significant rise in level of NOS, COX-2, NF-κβ, and TNF-α was assessed using a specific ELISA kit in ICV-STZ infused mouse brains in comparison to the normal group. Many reports were concurrent to our findings regarding the increased release of neuroinflammatory cytokines associated with STZ infusion in mice brain [67,88]. On the other hand, STZ-ICV mice treated with free LUT resulted in a significant reduction of NOS, COX-2, and TNF-α levels as shown in Figure 10D–F. In addition, a 0.6-fold inhibition of NF-κB signaling pathways was also observed (Figure 10G) that was involved in the pathological effects of Aβ. Consequently, such findings highlight the significant anti-inflammatory activity of LUT. Our findings were consistent with several previously reported studies regarding the ability of luteolin to reduce the release of inflammatory mediators induced by lipopolysaccharide-stimulated microglial cells [7,74,89].

##### Estimation of *Aβ1-42* and Tau

Many hypotheses were imposed speculating amyloid beta (Aβ) as a major pathological hallmark of AD [67]. In addition, Tau protein is hyperphosphorylated in AD and forms pathological accumulates in neurons which leads to learning and memory impairments [90]. Our results showed an elevation in *Aβ1-42* and Tau levels in mice injected with STZ-ICV compared to negative control group receiving ICV saline (Figure 10H,I). The reason was reported to be due to the fact that ICV-STZ injection decreases the cerebral glucose uptake, desensitizes brain insulin receptors, and consequently leads to tau hyper-phosphorylation [91]. Moreover, glucose hypo-metabolism initiates the process of Aβ aggregation [92]. On the other hand, the administration of free LUT (50 mg/kg, i.n) for 21 days led to a decline in the level of these peptides. The LUT-CHS altered the level of *Aβ1-42* and Tau peptides more potentially than its free form. Results demonstrated 0.53- and 0.54-fold reduction in the levels of *Aβ1-42* and Tau respectively, compared to the model control group. These may be attributed to the fact that luteolin has the potential to suppress Aβ and promote tau disaggregation [93]. These notions present that LUT-CHS was capable of attenuating the pathological changes of AD.

##### Estimation of MMP9

Increased MMP-9 expression is involved in the development of cognitive impairment induced by beta-amyloid [92]. Results demonstrated in Figure 10J showed a significant increase in MMP9 level in the STZ group compared to the normal mice group. On the other hand, results of free LUT exhibited a significant decrease in MMP9 levels which highlighted the mechanism of LUT concerning the down-regulation of the expression of MMP 9. Results were in harmony with Ali F and Siddique YH [93], who reported a decrease in permeability and infiltration of leukocytes and other inflammatory agents into the brain after LUT use. Furthermore, the LUT-CHS showed a higher decline in MMP9 level in brain tissue with no significant difference relative to the normal group. Such effects may be referred to the role of nanoplatforms in improvement of therapeutic efficacy of administered flavonoids.

##### Estimation of Transcription Factors (CREB)

Mouse CREB is a vital transcription regulator in nerve cells. Therefore, any impairment in CREB signaling may play a crucial role in the development of AD [94]. In this context, our results were in agreement with those reported and demonstrated by a significant decrease in CREB level in STZ group compared to normal group. Regarding LUT treatment, it modulated the activities of transcription factor CREB in harmony with that reported by Ali F and Siddique YH [93]. Figure 10K demonstrates a significant increase in CREB level in free LUT group (group 3) with a more profound increase in LUT-CHS group (group 4).

#### 3.5.5. Safety Studies

Visual observation of the animals showed that LUT suspensions showed no signs of nasal irritation with no considerable difference between placebo and drug loaded formulations confirming being cilio-friendly [4]. Recent studies identified that luteolin attenuates allergic nasal inflammation via inhibition of IL-4 production, which supports the potential pharmaceutical application of luteolin in nasal route. It should also be highlighted that using CS-NP as a drug delivery system offered optimum LUT encapsulation with sustained release that minimized the concentration of the drug in direct contact with nasal mucosa preventing nasal irritation [95].

It should be noted that the brain has a very narrow regenerative capacity [96]. Thus, brain targeted formulations should be assessed for their potential neurotoxic effects. Thus, the possible neurotoxic effect of the developed formulation was detected. The level of neural inflammatory reaction was also assessed by measuring TNF-” level in the hippocampus. As previously mentioned in “Estimation of pro-inflammatory mediators (NOS, COX-2, NF-κβ and TNF-α)” Section, STZ-ICV mice treated with free LUT resulted in a significant reduction of TNF-α levels. This finding highlights the significant anti-inflammatory activity of LUT. Further, based on the histological examination (Section 3.5.2), LUT-CHS showed apparently normal histological structure of the cerebral cortex with the best protective action against hippocampus damage in which all examined sections were apparently normal. It also showed an increased rate of neuronal survival rate. Thus, both visual observations of animals taking i.n. LUT loaded chitosomes did not reveal any inflammatory response and results of TNF-” level analysis and histological examination affirmed these observations. 

## 4. Conclusions

The current study presents novel LUT-loaded in chitosan-decorated nanoparticle (chitosomes) for the management of cognitive dysfunction in Alzheimer’s disease (AD). Multifaceted mechanisms of action were proven throughout the study with recorded significant improvement in short-term and long-term spatial memory. Histological evaluation revealed an increased neuronal survival rate with a reduction in the number of amyloid plaques. Biochemical results showed improved antioxidant effects and reduced pro-inflammatory mediators’ levels. The capability of luteolin-loaded chitosomes in attenuating the pathological changes of AD was also confirmed. The developed chitosomes offered the privileges of non-invasive intra-nasal delivery, lowering the administered dose with higher efficacy than the corresponding suspension form. Consequently, the proposed formulation ameliorates the impairments in cognitive function, and is thus a promising therapeutic tactic in the management of SAD.

## Figures and Tables

**Table 1:** The composition of various formulations of empty and Luteolin-loaded anionic liposomes, and chitosan coated liposomes (Chitosomes) prepared by ethanol injection method.

Ingredients (%*w*/*v*)	F1	F2	F3	F4	F5	F6
Phosphatidyl choline	0.5	0.5	0.5	0.5	0.5	0.5
Cholesterol	25	25	25	25	25	25
Phosphatidyl serine	3	3	3	3	3	3
Luteolin	-----	20 ^a^	40 ^b^	20	20	20
Chitosan	-----	-----	-----	2	4	8

^a^ Luteolin dose equivalent to 50 mg/kg. ^b^ Luteolin dose equivalent to 100 mg/kg.

**Table 2:** Mean Particles Size, Polydispersity Index, Zeta Potential and entrapment efficiency of empty liposomes, luteolin loaded liposomes, empty chitosomes and Luteolin-loaded Chitosomes. Measurements are expressed as mean ± SD.

Formulation Code	Particle Size (nm) ± SD	PDI± SD	Zeta Potential (mV) ± SD	Entrapment Efficiency% ± SD
F1 (Empty Lip.)	184.6 ± 1.85	0.3014 ± 0.06	−24.9 ± 4.21	NA
F2 (Lip-LUT_20 a_)	212.3 ± 2.18	0.398 ± 0.09	−33.1 ± 2.11	80.6 ± 1.28
F3 (Lip-LUT_40 b_)	320.0 ± 4.23	0.407 ± 0.25	−31.6 ± 1.48	77.9 ± 1.33
F4 (LUT-CHS_2% c_)	347.2 ± 1.84	0.419 ± 0.04	28 ± 2.94	79.2 ± 2.54
F5 (LUT-CHS_4% d_)	412.8 ± 3.28	0.378 ± 0.07	37.4 ± 2.13	86.6 ± 2.05
F6 (LUT-CHS_8% e_)	473.1 ± 2.14	0.274 ± 0.03	36.2 ± 3.04	81.5 ± 1.77

^a^ Luteolin load equivalent to 20 mg%, ^b^ Luteolin load equivalent to 40 mg%, ^c–e^ Luteolin-loaded chitosomes with different chitosomes concentrations (2, 4, 8 mg%. respectively) and luteolin loading of 20 mg%.

**Table 3:** Stability Study for LUT-CHS stored in refrigerator at 4 °C within 6 months.

Months	Size (nm)	PDI	Zeta (mv)	EE (%)
0	412.8 ± 3.28	0.378 ± 0.07	37.4 ± 2.13	86.6 ± 2.05
3	426.1 ± 3.01	0.400 ± 0.11	35.8 ± 3.17	85.1 ± 3.01
6	435.3 ± 2.81	0.415 ± 0.56	34.3 ± 3.04	82.8 ± 1.15

**Table 4:** Parameters of the different kinetic models for the LUT-CHS.

Model Name and Equation	Parameters	Values
Higuchi * F = k_H_ √t	Rsqr_adj	0.909
	MSE	94.59
	k_H_	21.38
	MSC	1.86
Hixson-Crowell * $1-\sqrt[{3}]{1-F}=k1/3t$	Rsqr_adj	0.975
	MSE	27.49
	k_1/3_	0.043
	MSC	3.09
Korsmeyer-Peppas * $F=k_{P}*t^{n}$	Rsqr_adj	0.900
	MSE	103.7
	n	0.462
	k_p_	23.43
	MSC	1.68
Weibull * $F=100*(1-e^{-{\left(t\right)}^{\beta }/\alpha }$)	Rsqr_adj	0.992
	MSE	16.49
	β	0.900
	MSC	3.478

* In all models, F denotes the fraction (%) of drug released up to time t, kH, k_1_, kp: release constants of Higuchi, first order, Korsmeyer-Peppas respectively, n: release exponent α: is the scale parameter, *β*: the shape parameter, Rsqr_adj; adjusted coefficient of determination, MSC; Model Selection Criterion and MSE; the mean square error.

**Table 5:** The number of amyloid plaques recorded in the brain of the mice.

Number of Amyloid Plaques (High Microscopic Field)	
Group (1)	ــــــــــــــــــــــ
Group (2)	6.2 ± 0.41 ^c^
Group (3)	2.6 ± 0.4 ^b^
Group (4)	1.4 ± 0.26 ^a^

Data were expressed as means ± SD. a, b and c indicate significant difference within the same column. Significant difference is considered at *p* < 0.05.

## Data Availability

Not applicable.

## Supplementary Materials

The following supporting information can be downloaded at: https://www.mdpi.com/article/10.3390/pharmaceutics14051003/s1, Figure S1: Chemical structure of luteolin, Figure S2: Particle size distribution (PSD) of LUT-CHS.
