# Supplementary material for: Novel Luteolin-Loaded Chitosan Decorated Nanoparticles for Brain-Targeting Delivery in a Sporadic Alzheimer’s Disease Mouse Model: Focus on Antioxidant, Anti-Inflammatory, and Amyloidogenic Pathways
Source: Pharmaceutics. 2022 May 6;14(5):1003. doi: 10.3390/pharmaceutics14051003 (PMC9148113; doi:10.3390/pharmaceutics14051003)
Supplement: Supplementary file 1 [file pharmaceutics-14-01003-s001.zip › pharmaceutics-1691512-supplementary.pdf]

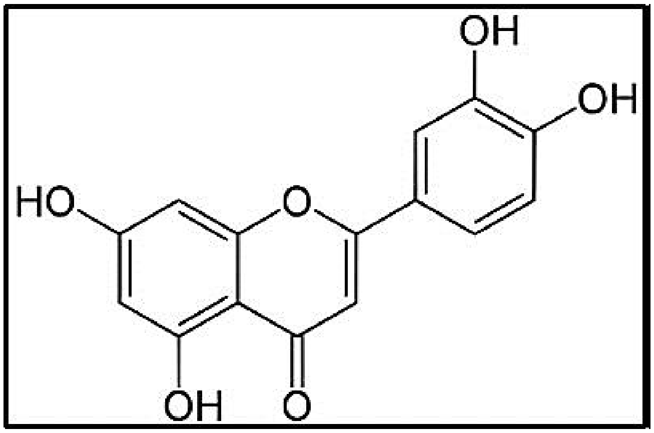

Figure S1

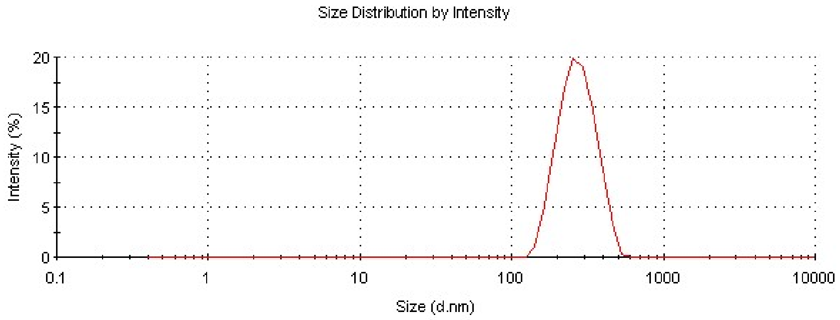

Figure S2

Figure S1 Chemical structure of luteolin

Figure S2 Particle size distribution (PSD) of LUT-CHS
